# Editorial: Adiponectin: Friend or Foe? Toward Understanding the Complexities of Adiponectin Biology and Challenges in Pharmaceutical Development

**DOI:** 10.3389/fendo.2020.00343

**Published:** 2020-05-29

**Authors:** Eva Surmacz, Tania Fiaschi

**Affiliations:** ^1^Allysta Pharmaceuticals, Inc., Belmont, CA, United States; ^2^Department of Biomedical, Experimental and Clinical Sciences, University of Florence, Florence, Italy

**Keywords:** adiponectin, adiponectin agonists, cancer, Alzheimer's, cardiovascular disease, adiponectin paradox

This topic collection focuses on adiponectin, one of the major adipokines. Adiponectin has been first described as a beneficial cytokine exhibiting insulin-sensitizing, anti-inflammatory, anti-atherogenic activities, and even anti-cancer properties. Numerous *in vitro* and animal studies continue to validate these claims. However, new evidence also suggested that, paradoxically, elevated adiponectin levels can be associated with induction or existence of some pathologies.

We strongly believe that research and medical communities interested in adiponectin will benefit from this updated discussion tackling the multifaceted nature of this protein and the challenges associated with a potential pharmaceutical development. This collection of peer-reviewed articles highlights the complexity of adiponectin biology, focusing on different diseases and/or sites of action, including cardiovascular disease, neurological disorders, and cancer development. Several reviews explore the utility of adiponectin as biomarker and biotarget as well as discuss strategies to rebalance adiponectin levels using novel pharmacological entities.

Adiponectin action is greatly impaired in cardiovascular diseases due to the reduced bioavailability and/or target tissue resistance. Consequently, the development of strategies to counteract hypoadiponectinemia in these pathologies is of great interest. The review of Liu et al. “*Examining the Potential of Developing and Implementing Use of Adiponectin-Targeted Therapeutics for Metabolic and Cardiovascular Diseases “* describes the physiological effects of adiponectin in cardiac tissue and potential impact of this adipokine in cardiovascular diseases. Furthermore, the authors suggest the use of adiponectin as a biomarker and a new tool for the detection and management of cardiovascular pathologies. Along these lines, a possibility to alleviate adiponectin deficiency in cardiac pathologies by employing adiponectin receptor agonists (i.e., AdipoRon, osmotin, ADP355, ADP399) is discussed.

Epidemiological studies suggest that excessive perirenal fat increases the risk of cardiometabolic risk, hypertension, and cardiovascular disease. Maimaituxun et al. in the article “*Levels of Adiponectin Expression in Peri-Renal and Subcutaneous Adipose Tissue and Its Determinants in Human Biopsied Samples”* address the role of perirenal fat adipocytokines, especially adiponectin, in kidney pathologies. The authors evaluated adiponectin expression in perirenal adipose tissue (RAT), visceral adipose tissue (VAT), and subcutaneous adipose tissue (SAT) in urological surgery patients. Levels of adiponectin mRNA in RAT were found to be negatively correlated with remote fat mass in SAT and VAT, and with local fat mass in RAT, while levels of adiponectin in SAT were not associated with RAT volume. The authors discuss a possible role of RAT adiponectin and RAT volume in kidney function as well as in local and systemic inflammation.

Growing evidences suggest a protective role of adiponectin in several brain disorders including Alzheimer's disease, stroke, and depression. Although adiponectin receptors, AdipoR1 and AdipoR2, are widely distributed in various brain regions, the involvement of adiponectin in specific brain activities is not well-understood. The results presented by Bloemer et al. in the paper “*Adiponectin Knockout Mice Display Cognitive and Synaptic Deficits*” support an important role of adiponectin in brain functionality. Using adiponectin knockout mice, the authors demonstrated that adiponectin deficiency impairs synaptic processes and cognitive function. Most importantly, the knockout animals exhibited cognitive deficits, reduced basal synaptic transmission, increased presynaptic release probability, impaired synaptic plasticity, and altered glutamatergic receptor levels.

In the nervous system adiponectin appears to display both beneficial and toxic effects. Indeed, some studies performed in cellular and animal models reported a neuroprotective role of adiponectin, while others show a correlation between adiponectin, cognitive decline and severity of amyloid accumulation in elder population. Waragai et al. in their article **“***Adiponectin Paradox in Alzheimer's Disease; Relevance to Amyloidogenic Evolvability***”** focus on the adiponectin paradox in Alzheimer's disease (AD) and discuss potential mechanisms involved. It is suggested that adiponectin might be involved in the stimulation of the amyloidogenic evolvability in reproductive stages, which may later manifest as AD by the antagonistic pleiotropy during aging. The authors also speculate on the potential links between the adiponectin paradox in AD and similar unusual adiponectin effects seen in other advanced age diseases such as chronic heart failure and chronic kidney disease.

Numerous studies highlighted the potential role of adiponectin in cancer development and progression. Especially, clinical data indicate that low adiponectin levels are associated with increased risk and more aggressive phenotype in breast cancer, while higher levels might have a protective effect. However, experimental data appear to suggest a more complex situation. The review of Naimo et al. ‘*Interfering Role of ER*α *on Adiponectin Action in Breast Cancer”* discusses the role of adiponectin in breast cancer depending on estrogen receptor alpha (ERα) status. The authors argue that in obese patients, adiponectin may, paradoxically, act as a growth enhancer for ERα-positive breast cancer cells. Based on *in vitro* and *in vivo* evidence, this response could be related to ERα signaling overriding the inhibitory action of adiponectin. In contrast, in the absence of ERα, adiponectin may exhibit anti-neoplastic activities. The molecular mechanisms underlying the adiponectin/ERα interface are discussed in detail.

A large case-control clinical study presented by Xiang et al. and its addendum “*Metabolic Syndrome, and Particularly the Hypertriglyceridemic-Waist Phenotype, Increases Breast Cancer Risk, and Adiponectin Is a Potential Mechanism: A Case–Control Study in Chinese Women*” provides an interesting analysis suggesting that low adiponectin levels in conjunction with metabolic syndrome, especially the hyperglyceridemic waist phenotype, can be regarded as a strong predictor of breast cancer in Chinese women.

The compelling experimental and clinical evidence strongly supports the efforts to develop clinically viable compounds targeting adiponectin signaling in human pathologies. Due to pharmacological disadvantages of the intact adiponectin protein, potential hypoadiponectinemia treatments focus on the use of peptidic, and small molecule agonists of the adiponectin receptor. A comprehensive review by Otvos “*Potential Adiponectin Receptor Response Modifier Therapeutics*” details currently tested peptide-based adiponectin replacement drug leads as well as discusses pharmaceutical potentials of small molecule therapies. The author analyzes advantages and difficulties in the reliability and readout of the *in vitro* measures and the wealth of *in vivo* models that make comparison of the various drug classes complicated. Alternative approaches to direct adiponectin signaling control that use adiponectin-upregulating strategies but offer relatively limited success compared to direct receptor agonists are also evaluated.

## Author Contributions

ES and TF wrote and edited the editorial.

## Conflict of Interest

ES serves as Senior Advisor for Allysta Pharmaceuticals, a company developing adiponectin-based therapeutics. The remaining author declares that the research was conducted in the absence of any commercial or financial relationships that could be construed as a potential conflict of interest

